# Economic pressures of Covid‐19 lockdowns result in increased timber extraction within a critically endangered region: A case study from the Pacific Forest of Ecuador

**DOI:** 10.1002/ece3.9550

**Published:** 2022-11-22

**Authors:** Jacquelyn M. Tleimat, Sarah R. Fritts, Rebecca M. Brunner, David Rodriguez, Ryan L. Lynch, Shawn F. McCracken

**Affiliations:** ^1^ Department of Life Sciences, College of Science and Engineering Texas A&M University – Corpus Christi Corpus Christi Texas USA; ^2^ Department of Biology, College of Science and Engineering Texas State University – San Marcos San Marcos Texas USA; ^3^ Department of Environmental Science, Policy, and Management, College of Natural Resources University of California – Berkeley Berkeley California USA; ^4^ Third Millennium Alliance Fremont California USA

**Keywords:** anthropause, COVID‐19 repercussions, deforestation, environmental crime, passive acoustic monitoring

## Abstract

Although the COVID‐19 lockdowns in 2020 had some environmental benefits, the pandemic's impact on the global economy has also had conservation repercussions, especially in biodiverse nations. Ecuador, which is heavily reliant on petroleum, agricultural exports, and ecotourism, experienced a rise in poverty in response to pandemic shutdowns. In this study, we sought to quantify levels of illegal timber extraction and poaching before and after the start of COVID‐19 lockdowns throughout two protected areas (Reserva Jama Coaque [JCR] and Bosque Seco Lalo Loor [BSLL]) in the endangered Pacific Forest of Ecuador. We analyzed chainsaw and gunshot acoustic data recorded from devices installed in the forest canopy from December 2019 to March 2020 and October 2020 to March 2021. Results from generalized linear mixed effects models indicated less chainsaw activity before lockdowns (*β*post.lockdown = 0.568 ± 0.266 SE, *p*‐value = .030), although increased average rainfall also seemed to negatively affect chainsaw activity (*β*avg.rainfall = −0.002 ± 0.0006 SE, *p*‐value = .003). Gunshots were too infrequent to conduct statistical models; however, 87% of gunshots were detected during the ‘lockdown’ period. Observational data collected by rangers from these protected areas also noted an increase in poaching activities beginning mid to late 2020 and persisting into 2021. These results add to the steadily growing literature indicating an increase in environmental crime, particularly in biodiverse nations, catalyzed by COVID‐19‐related economic hardships. Identifying areas where environmental crime increased during pandemic lockdowns is vital to address both socioeconomic drivers and enforcement deficiencies to prevent further biodiversity loss and disease outbreaks and to promote ecosystem resilience. Our study also demonstrates the utility of passive acoustic monitoring to detect illegal resource extraction patterns, which can inform strategies such as game theory modeling for ranger patrol circuits and placement of real‐time acoustic detection technologies to monitor and mitigate environmental crimes.

## Graphical abstract

Although COVID‐19 lockdowns have had some environmental benefits, the impact on the global economy has also led to conservation repercussions. In our study, we analyzed bioacoustic data from the canopy of the Pacific Forest of Ecuador to compare chainsaw activity before and after the start of COVID‐19 lockdowns. Our results indicate that COVID‐19 lockdowns were associated with an increase in chainsaw activity.

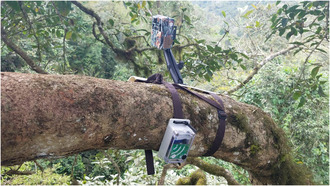

## 1 INTRODUCTION

In March 2020, the SARS‐CoV‐2 outbreak led to lockdowns that drastically reduced human travel and economic activity (Bates et al., 2020), which inadvertently impacted wildlife and their habitats. One of the most publicized effects was increases in novel sightings of wildlife in urban and peri‐urban areas (Silva‐Rodríguez et al., 2021) and increased sightings of ‘human‐avoidant’ species (Rutz et al., 2020; Wilmers et al., 2021). Outside of urban areas, reported decreases in human activity in several natural areas positively impacted some species due to reduced disruption during reproduction events (Corlett et al., 2020; Rutz et al., 2020; Schofield et al., 2021). Additionally, decreased vehicular traffic on roads and waterways temporarily decreased air and water pollution in many places (Bao & Zhang, 2020; Dantas et al., 2020; Faridi et al., 2021; Kerimray et al., 2020; Saraswat & Saraswat, 2020) and reduced wildlife–vehicle collisions (Bíl et al., 2021; LeClair et al., 2021; Shilling et al., 2021). Collectively, this period of lockdowns was termed by researchers as the ‘anthropause’ (Rutz et al., 2020). The dominating narrative of the ‘anthropause’ seems to be focused on the effects of decreased human impacts on the environment and speculations on how resource extraction may change in response to these drastic changes.

However, not all countries experienced a uniform decline in human–nature interaction; increases in environmental crime due to lockdowns were reported in several countries (Price, 2020). For example, spikes in poaching were reported more frequently (Aditya et al., 2021; Koju et al., 2021; Price, 2020; Rahman et al., 2021), a greater emphasis was placed on wildlife trade (Morcatty et al., 2021), and satellite imagery revealed an increase in deforestation across several tropical countries (Brancalion et al., 2020; Rahman et al., 2021) facilitated by higher unemployment—especially of park rangers. Unfortunately, much of these data were confined to news reports which limit the ability to understand true trends given the historic underreporting of environmental crime (Barclay & Bartel, 2015) coupled with lowered employment of park rangers during this period. To fully assess the impact of the ‘anthropause’, it is evident that the evaluation of datasets from countries that were financially impacted the most by the pandemic is a necessity.

The economy of Ecuador, a megadiverse country in South America, was largely driven by petroleum, agriculture, and ecotourism before the COVID‐19 pandemic; thus, it was severely impacted by lockdowns as oil demand and tourism declined (Heritage Foundation, 2022; Lara, 2022). In fact, GDP decreased by nearly 8%, resulting in the poverty threshold currently encompassing 30% of Ecuador's population (ECLAC, 2021), which exacerbated economic inequality and the consequent social issues. This economic crisis coupled with declines in ability to enforce human restrictions within protected areas (Price, 2020) likely motivated individuals to seek illegal resource extraction as a source of income, especially when presented with the emerging market created by the boom in wind energy. Specifically, increases in wind energy created an increase in demand for balsa wood (*Ochroma pyramidale*), which comprises ~2.3% of a typical wind turbine blade by weight (Liu & Barlow, 2016), and then a concomitant rise in balsa prices. Job insecurity and loss, decreased ability to protect forests, and increase in demand of wind energy, together, have created a dynamic that benefits individuals who pursue illegal logging. Although reports have been made on the drastic increase in illegal logging as of 2020, the Amazon has been the primary focus (Baquero, 2021) despite the existence of logging throughout Ecuador (Tomaselli, 2019).

The Pacific Forest of Ecuador is a greatly imperiled ecoregion (Dodson & Gentry, 1991) that has lost 96% of its primary forest, largely due to slash‐and‐burn agriculture (Haro‐Carrión & Southworth, 2018). The export of timber such as teak, balsa, and gmelina (Tomaselli, 2019) has further contributed to this deforestation. From 2018 to 2020, the amount of advertising along Manabí Province roads for balsa purchasing increased noticeably (Pers. Obs.). Yet, despite the increased market for balsa and massive deforestation, this area of Ecuador has largely been ignored in reports of logging in the country. As such, there have been no quantitative reports on how the lockdowns have impacted timber extraction in Manabí Province despite being at the heart of the Tumbes‐Chocó‐Magdalena biodiversity hotspot (Mittermeier et al., 2011). Our objective was to compare chainsaw and gunshot activity in a protected area in the Pacific Forest of Ecuador before and after the start of COVID‐19 lockdowns by using passive acoustic devices that were opportunely installed months prior to the start of the pandemic. We hypothesized an increase in chainsaw and gunshot activity after the start of the COVID‐19 lockdowns, likely due to the ensuing economic crisis. This case study will add to the growing literature analyzing the effects of lockdowns on environmental crime and aid in directing appropriate conservation action. In the face of the COVID‐19 pandemic, a shift in conservation efforts toward a multidisciplinary approach (e.g., ‘One Health’) where human, environmental, and wildlife health are intrinsically linked (Zinsstag et al., 2005; Zinsstag & Tanner, 2008) are essential to mitigate future disease outbreaks.

## 2 METHODS

This study was located in the Pacific Forest of Ecuador within the Hacienda Camarones Key Biodiversity Area (HCKBA), located approximately 11 km south of the equator in the Manabí Province. The HCKBA encompasses 4000 ha and includes Reserva Jama Coaque (JCR; −0.116128, −80.124546), Reserva Bosque Seco Lalo Loor (BSLL; −0.077115, −80.153496), and the Three Forest Conservation Corridor which consists of the forested ridgeline between the two ecological reserves. This key biodiversity area is made up of 70% forest with the rest of the landscape encompassing pasturelands and urban areas. The climate is defined by a marked dry season from June through December and an intense rainy season from January through May (Dodson & Gentry, 1991). Rangers have been patrolling throughout the HCKBA for many years prior to the COVID‐19 pandemic and continue to patrol this protected area. There are no allowances of poaching or timber extraction throughout the HCKBA.

**FIGURE 1:**
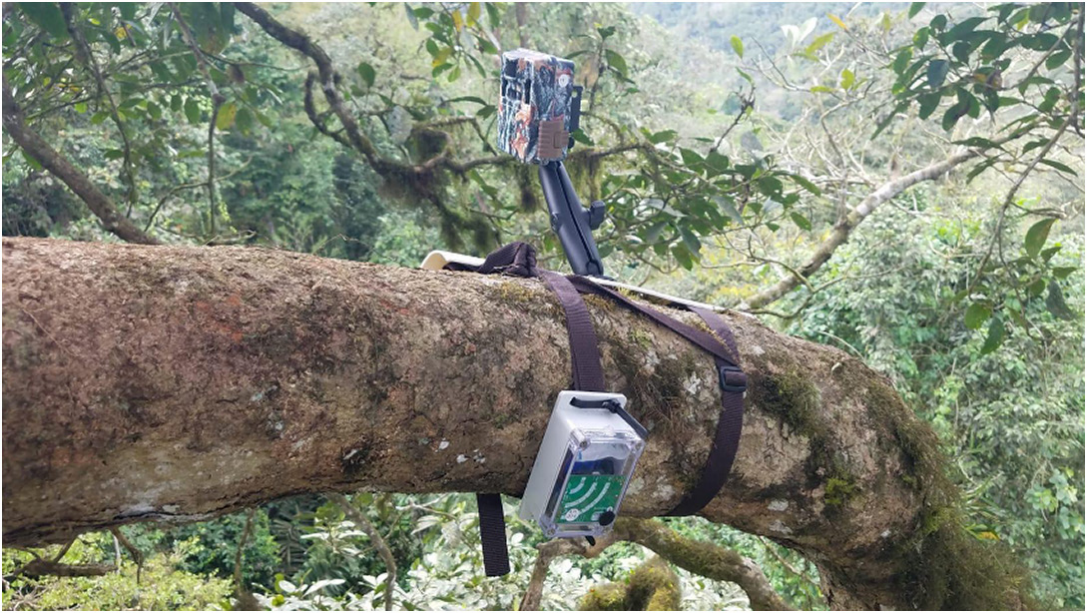
Image of a canopy station. The AudioMoth is positioned slightly under the tree to decrease the chance of water buildup and in a weatherproof case that has a membrane to allow sound to pass and a silica packet in the event moisture enters.

We monitored chainsaw and gunshot activity throughout the HCKBA using 18 AudioMoth (version 1.1, Open Acoustic Devices, United Kingdom) passive acoustic devices systematically spaced (distance to closest recorder; Min: 0.63 km, Max: 1.31 km, Avg: 0.95 km) and mounted (Figure 1) in the canopy (Min: 4 m, Max: 29.5 m, Avg: 16.6 m). We enclosed AudioMoths in waterproof electrical junction boxes with a hole drilled in the cover and a waterproof acoustic membrane adhered externally, with the microphone mounted flush on the inside of the cover (Figure 1). We then soldered a JST‐style power cable to the AudioMoth circuit board to allow the use of a 3.7 V 4400 mAh lithium‐ion battery pack (Adafruit Industries). In the canopy of each tree, we secured an AudioMoth that was set to record with a medium gain at a sampling rate of 48 kHz for 10 min every hour between 0600 and 1900 h. We completed establishment of the canopy stations on 5 January 2020 and data collection for this analysis on 25 March 2021.

We identified illegal activity using parameters that reflect vibrations created by chainsaws and gunshots identified in the literature and adjusted based on the acoustic detections produced by Kaleidoscope Pro (version 5.19, Wildlife Acoustics). We used the following parameters for chainsaws: 80–550 Hz frequency range, 0.5–20 s vocalization length, and 0.45 s maximum inter‐syllable gap (Matache et al., 2020). For gunshots, we used 200–2500 Hz frequency range, 0.001–0.75 s vocalization length, and 0.01 maximum inter‐syllable gap. We manually identified sound waves that Kaleidoscope Pro produced within the specified parameters. We created a proxy of chainsaw activity by creating a daily history of detected/non‐detected status. To ensure duplicate detections were not included in the analysis, we compared detection characteristics (time of the signal, chainsaw buzzing length, etc.) to determine if the sounds were produced by the same source. If two detections were determined to be from the same source, we only included the detection at the location it was loudest. We did the same for gunshot detections.

We conducted a generalized linear mixed effects model using the ‘glmmTMB’ package (Brooks et al., 2017), given the response variable (daily history of chainsaw activity coded as ‘1’ for detected, ‘0’ for non‐detected) was non‐normally distributed and the mix of fixed and random effects. We used two time periods related to COVID‐19 lockdowns: pre‐COVID‐19 lockdowns defined as December 2019–April 2020 and during/post‐COVID‐19 lockdowns defined as October 2020–March 2021 (these periods defined given the lack of data from May 2020 to early October 2020) as a categorical independent fixed effect, average monthly rainfall (mm) as a continuous fixed effect, and canopy station as a random effect. We included average monthly rainfall as it was likely to impact the ability to run a chainsaw. We compared a Poisson distribution to a zero‐inflated Poisson distribution using the lowest AICc to determine which best fit the data. We compared the best model to a null model using an ANOVA to ensure that variables explained variation in the dataset. We used a Wald test from the package ‘Activity’ (Rowcliffe et al., 2014) to compare the hourly chainsaw activity patterns between the time periods (i.e., whether peak times shifted). We conducted analyses in R version 4.1.1 (R Team, 2021; Rstudio 2021). We generated a heatmap of change in chainsaw frequency in response to lockdowns using QGIS (version 3.14, QGIS Development Team).

## 3 RESULTS

Chainsaw noises were detected across 14 canopy stations over 167 days: 39 days ‘pre‐lockdown’ and 128 days ‘during/post‐lockdown’. Gunshots were detected across five canopy stations over 8 days, seven in the ‘post‐lockdown’ period, and one in the ‘pre‐lockdown’ period. Gunshots were not used in any further analysis given the low detection.

**FIGURE 2:**
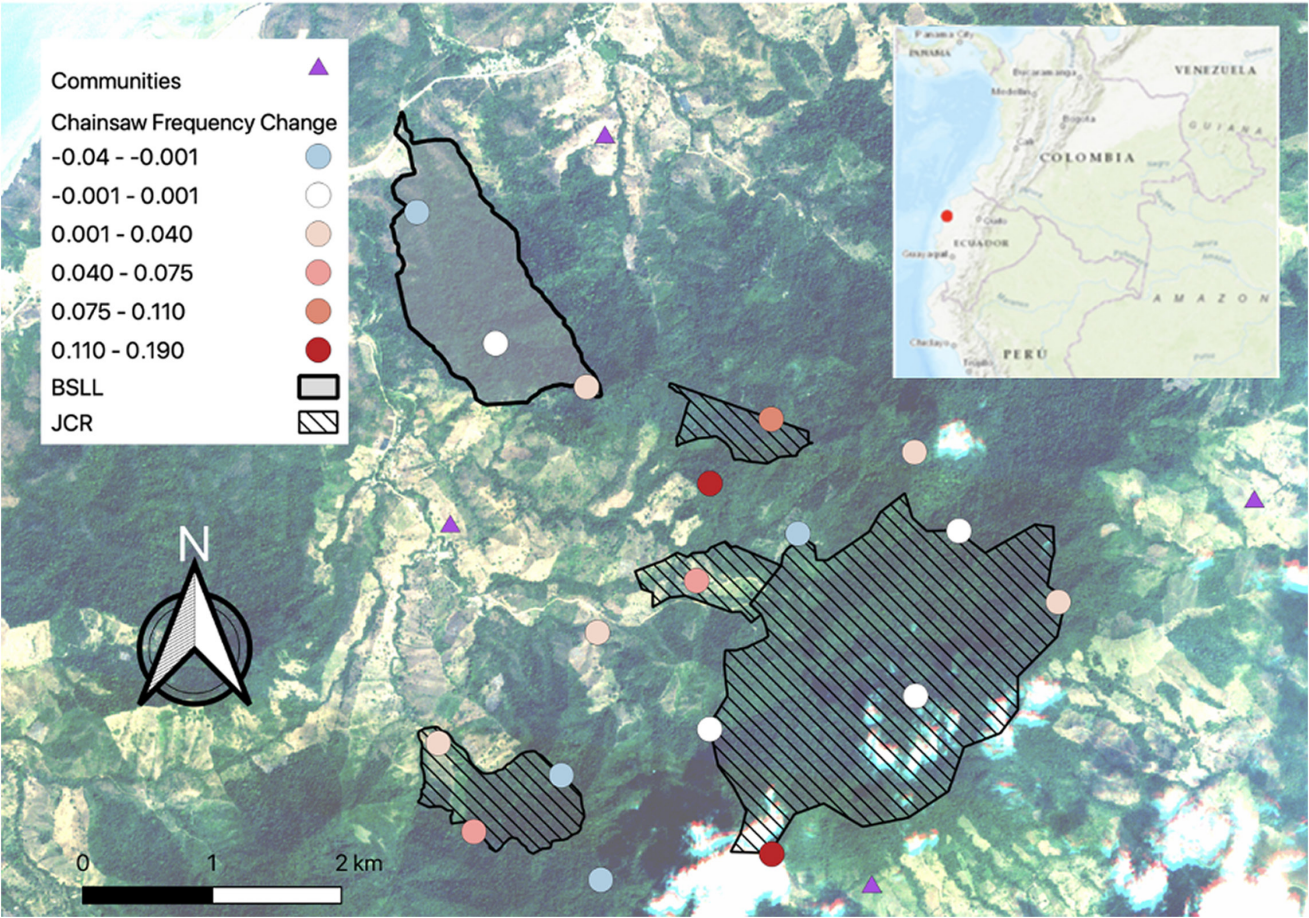
Change in frequency of chainsaw detections in the hacienda Camarones key biodiversity area (HCKBA) after COVID‐19 lockdowns (calculated by dividing total chainsaw detections per treatment by operating days and subtracting post‐Covid from pre‐Covid). Blue circles indicate a decrease in frequency, white indicates no change, and red indicates different degrees of increase. The outlined area filled with diagonal lines represents Reserva Jama Coaque (JCR), and the outlined shaded area represents Bosque Seco Lalo Loor (BSLL). Inset is a map of South America, provided by DataBasin, with the red circle indicating the location of the HCKBA along the northeastern coast of Ecuador.

**FIGURE 3:**
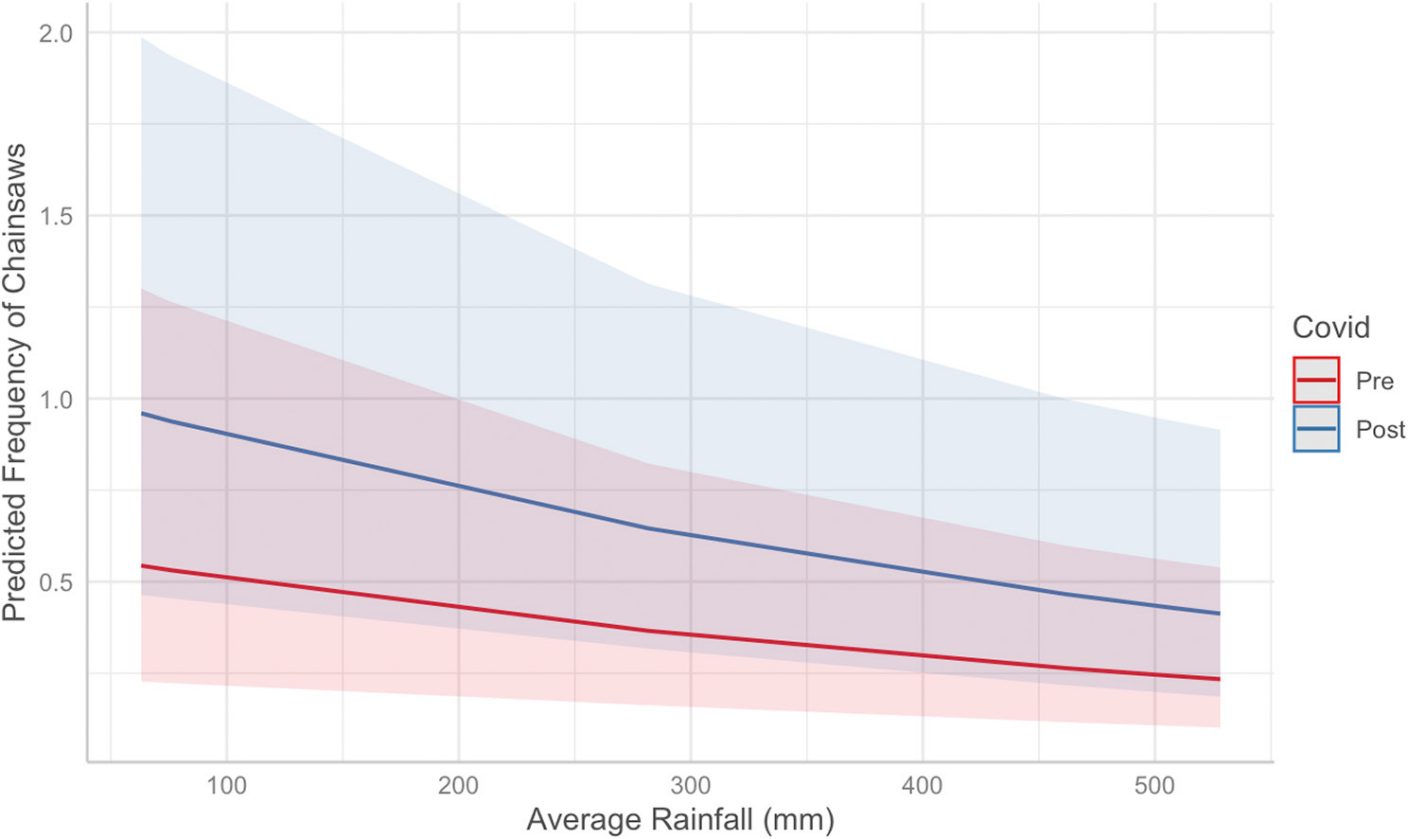
The predicted monthly chainsaw detection frequency by site based on the best fit model within the hacienda Camarones key biodiversity area in response to average rainfall (mm) by COVID‐19 lockdown period with 95% confidence intervals (shading). The frequency of chainsaw detections is predicted to decrease as monthly average rainfall decreases; however, it is also predicted to be higher after the start of COVID‐19 lockdowns (blue) compared to before the start of COVID‐19 lockdowns (red).

The number of chainsaw days varied between the time periods and was based on rainfall (Chisq = 334.200, *df* = 3, *p*‐value < .001). Based on AICc values, the best fit distribution for the GLMM was Poisson with log link distribution with the next AICc best model having Δ2.151. During the ‘post‐lockdown’ period, there was a 0.568 greater log count of expected chainsaws than in the ‘pre‐lockdown’ period (Figure 3) meaning that there were 3.698 greater chainsaw counts per site during the ‘post‐lockdown’ period compared to the ‘pre‐lockdown’ period. A one‐unit increase in average rainfall was associated with a 0.002 unit decrease in the expected log count of chainsaws (Figure 3). There was a high variance between sites (1.453 ± 1.206 SE), which is unsurprising given chainsaws were not detected at 23% of sites (Figure 2).

**FIGURE 4:**
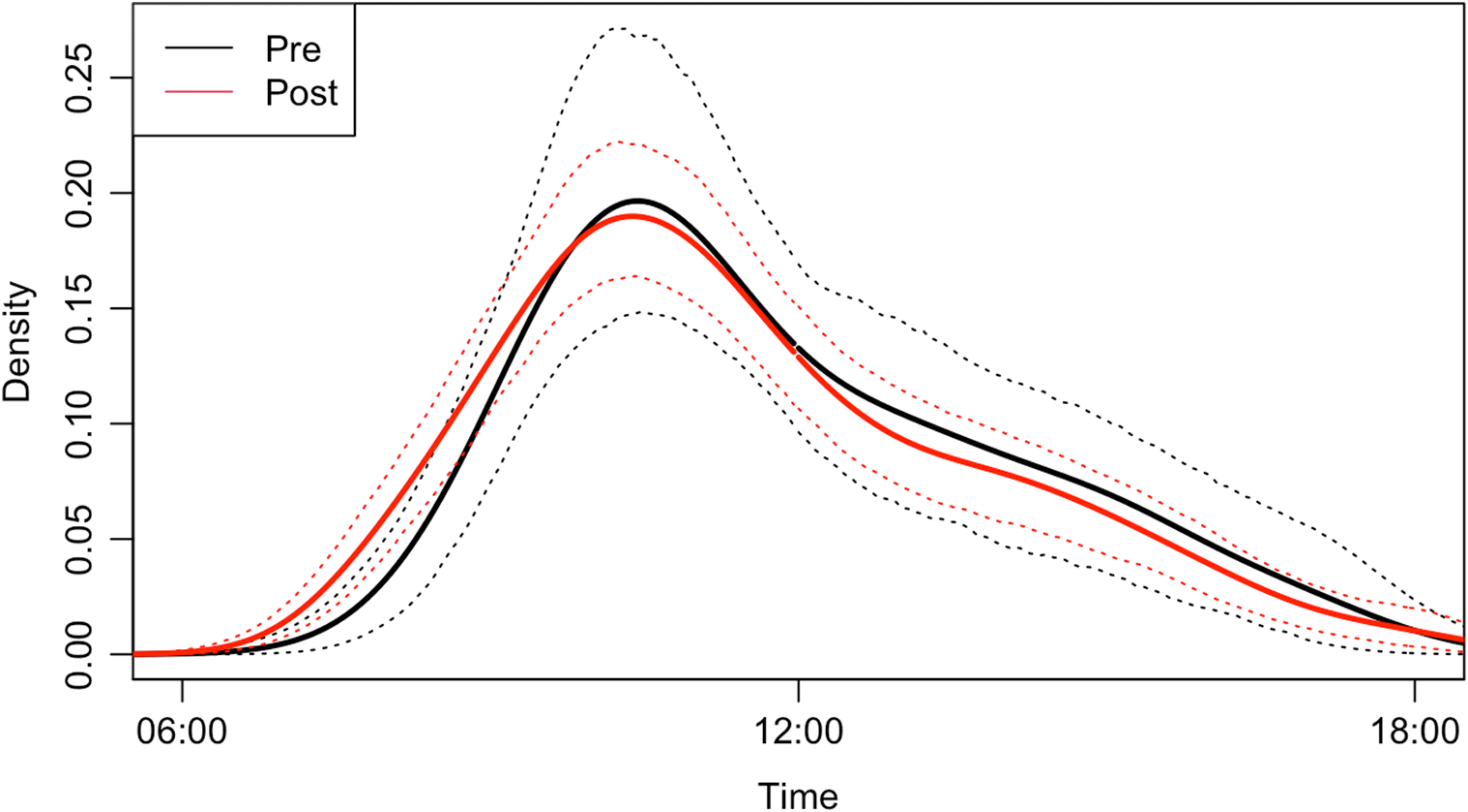
Hourly chainsaw activity before and after the start of COVID‐19 lockdowns in the hacienda Camarones key biodiversity area based on a 24‐h day. Chainsaw activity was only monitored from 0600 to 1800 h. The line in red represents chainsaw activity after the start of COVID‐19 lockdowns (post) and the dashed red lines represent the 95% confidence intervals. The line in black represents chainsaw activity before the start of COVID‐19 lockdowns (pre) and the dashed black lines represent the 95% confidence intervals. The peak of activity occurred around 10:00, and between the two time periods, there was no significant shift in time of activity.

The activity curves based on bootstrapped data of each COVID‐19 period did not differ based on a Wald test (Diff: −0.007, SE: 0.033, *p*‐value: .843), indicating no change in time of day in chainsaw activity as a result of the COVID‐19 lockdowns (Figure 4). The activity curve illustrates a daily peak in chainsaw activity from 0900 to 1400 h. Although the passive acoustic devices only recorded from 0600 to 1900 (during daylight hours), no chainsaws were detected before 0800 or at 1900 h, so it is unlikely we failed to capture other hours of chainsaw activity.

## 4 DISCUSSION

Illegal extraction of timber resources by chainsaws within a protected area of the endangered Pacific Coastal ecosystem of Ecuador increased during and following COVID‐19 lockdowns. This pattern is supported by the increased law enforcement seizures of illegal balsa throughout Ecuador with a spike of 180% from 2019 to 2020 (Coba, 2021); though this rise was largely attributed to the balsa boom (Baquero, 2021), the economic crisis undoubtedly contributed to this spike in illegal balsa harvesting. Similarly, agricultural expansion and increases in illegal mining in neighboring Brazil and Colombia resulted in greater illegal resource extraction during and after COVID‐19 lockdowns (Duque, 2020; Iglesias, 2020). Our results also seem to suggest rainfall influenced chainsaw activity (Figure 3); however, this is likely an artifact of the sampling design given we did not have the data to compare many low rain months before lockdowns. Our study was conducted in response to a unique opportunity (i.e., a global pandemic affecting humans), and thus on a limited scale, yet it provides quantitative evidence that these resource extraction patterns were associated with COVID‐19 lockdowns.

**FIGURE 5:**
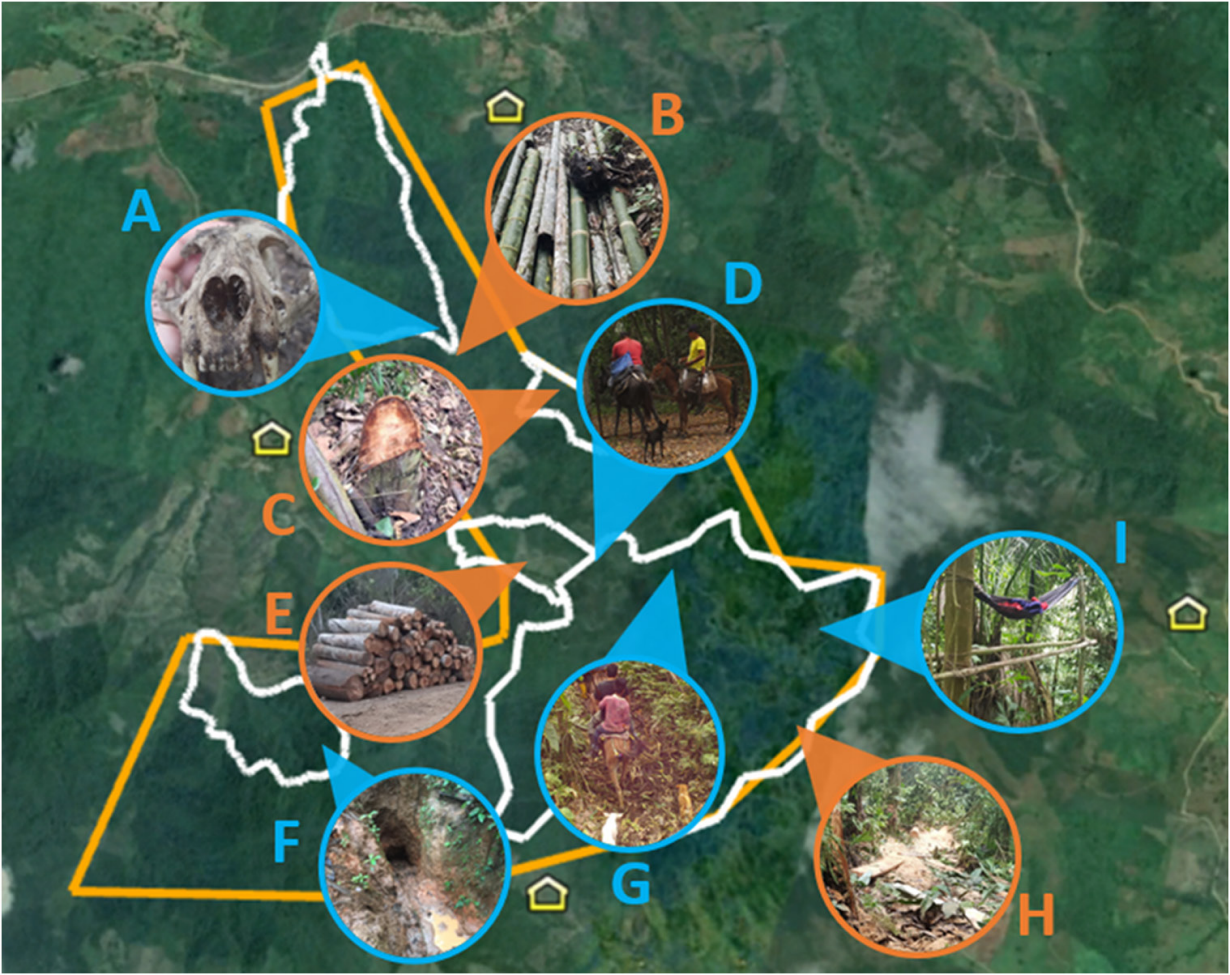
Map of known illegal activity throughout the hacienda Camarones key biodiversity area 2020–2021. Orange circles indicate evidence of timber extraction and blue circles indicate evidence of poaching. Yellow house icons indicate the location of communities. Each photo has been labeled for description of evidence. Photo a: Felid skull left on trail in protected reserve. Photo b: Bamboo stalks left to the side of the trail. Photo c: Stump of tree from protected area in the process of being cleared out. Photo d: Security trail camera photo of individuals entering the protected reserve on mules with machetes and collection bags. Photo e: Stack of balsa wood left by road for collection. Photo f: Natural clay pit that has been enlarged with evidence of use for human hunting. Photo g: Security trail camera photo of individuals carrying rifles on mules through protected reserve. Photo h: Saw dust and planks covering trail of protected reserve. Photo i: Campground set up by unknown individuals on protected reserve.

Even though limited data on gunshots were available throughout the protected areas we sampled, it is noteworthy that 87% of gunshot detection days were during the ‘post lockdown period.’ Rangers from BSLL have noted an increase in poaching activity as of October 2020, and similarly, increased poaching has been noted by JCR rangers as of summer of 2020 (Figure 5). Both security trail cameras and visual observations by rangers detected more frequent foot traffic in JCR (Pers. Comm.). Unfortunately, thorough records were not maintained until the increased traffic was observed for baseline comparison, but these records are now consistently maintained to monitor changes in foot traffic.

Patterns of illegal activity within the HCKBA will continue to be monitored with passive acoustic devices, especially following the implementation of shared agroforestry practices with local landowners to assess their effectiveness in reducing illegal resource extraction. Our results suggest that sites at the borders of property lines experienced the greatest increase in chainsaw activity (Figure 2), indicating that boundary lines should be patrolled more frequently. Spatial and temporal data from this study will be used to strengthen game‐theory models to inform patrolling regimes of the park rangers. Additionally, the temporal data from this study provide a potential guideline for surrounding reserves on ‘peak’ daylight extraction times (0900–1400 h) to aid in guiding their own patrol regimes.

Previous studies have demonstrated the effectiveness of using passive acoustic devices to monitor poaching activity and illegal vessel activity and adjust on the ground patrolling efforts (Astaras et al., 2020; Katsis et al., 2022; Kline et al., 2020; Piña‐Covarrubias et al., 2019; Prince et al., 2019), and our study indicates that these devices are also useful to monitor chainsaw activity. Given the limited number of gunshots detected, further research on appropriate recording intervals and live monitoring technology is needed to optimize gunshot detection. It is also worth noting that our device placement was not optimized for gunshot detection and our recording regime did not include nighttime hours, when illegal hunting often occurs. Further, the 10‐min recording window makes it likely that rare, short events, like gunshots, are missed. However, for conservationists and managers planning to use acoustic recorders to monitor hunting activity, probabilistic models have been developed to aid in an optimized placement of acoustic recorders across different terrestrial terrains (Piña‐Covarrubias et al., 2019). Additionally, the creation of detection algorithms and convolutional neural networks specifically for gunshots allows for relatively quick and accurate detection of gunshot events from big datasets (Katsis et al., 2022; Prince et al., 2019). With the cost‐effectiveness of passive acoustic devices and the development of advanced algorithms to hasten the process of event detection, we would recommend this method to other protected areas as a means to identify peak extraction times, activity hotspots, and set a chainsaw activity baseline to compare after logging regulations or patrolling regimes are changed (Astaras et al., 2020).

It is vital that further investigation on the impacts of COVID‐19 lockdowns on environmental crime continue, particularly in regions of high biodiversity, as disease outbreak and pandemic events are on the rise (Lindahl & Grace, 2015; Myers & Patz, 2009) and this information can help create safeguards to prevent similar environmental repercussions. Regions of high biodiversity, such as the Pacific Forest of Ecuador and the Amazon, have a greater potential for zoonotic spillover events (i.e., pathogen transmission from wildlife to humans), after a contact event (Daszak et al., 2000), compared to regions like the USA or Europe. For one, these biodiverse regions have high pre‐existing pathogenic diversity, which increases the probability of a pathogen with epidemic potential existing, and second, rising human–wildlife interaction in these areas increases the potential for contact events (Ellwanger et al., 2020). Although there are high standing levels of pathogen diversity in these regions, the maintenance of biodiversity supports a healthy ecosystem, in large part by keeping vector populations under control (Keesing et al., 2010); these controls are thrown out of equilibrium by deforestation and other such anthropogenic disturbances allowing vectors and pathogens to spread and occupy new niches (Aguirre & Tabor, 2008; Pongsiri et al., 2009). Often these anthropogenic disturbances leave forests in small, fragmented patches that may lead to a higher risk of spillover through the creation of independent ‘co‐evolutionary’ units, thus driving greater pathogen diversity (Zohdy et al., 2019). To protect the health of human populations and prevent further widespread pandemics, ecosystem health needs to be preserved and a key element of that is restricting environmental crime.

A growing number of researchers are calling to change to a ‘One Health’ perspective of conservation as continued environmental degradation presents a clear threat not only to wildlife but to human health as well (Beirne, 2021; Halabowski & Rzymski, 2021; Kadykalo et al., 2022; Kideghesho et al., 2021; Koju et al., 2021; Manenti & Hymas, 2021; Rahman et al., 2021; Scanlon AO, 2021; Zahawi et al., 2020). In this paper, we call for greater documentation of habitat loss and modification, particularly in highly biodiverse areas. Areas where environmental crime is increasing, should be identified to investigate and understand the main contributors; areas where logging industries are the main cause, should trigger effective changes in regulations; however, in other cases, economic safety nets and regenerative harvesting practices (such as agroforestry) should be established to protect struggling communities to remove the need for illegal resource extraction. While many researchers have called upon increased surveillance and stricter regulation on the logging industry, we believe it is also imperative to identify the socioeconomic drivers of environmental crime and seek solutions that eliminate the need for this source of income.

## AUTHOR CONTRIBUTIONS

**Jacquelyn Tleimat:** Conceptualization (equal); data curation (lead); formal analysis (lead); funding acquisition (equal); investigation (equal); methodology (supporting); validation (lead); visualization (equal); writing – original draft (lead); writing – review and editing (supporting). **Sarah R. Fritts:** Funding acquisition (equal); project administration (supporting); resources (equal); validation (supporting); visualization (equal); writing – original draft (equal); writing – review and editing (equal). **Rebecca M. Brunner:** Conceptualization (supporting); funding acquisition (equal); resources (equal); writing – original draft (supporting); writing – review and editing (equal). **Ryan L. Lynch:** Conceptualization (supporting); funding acquisition (equal); investigation (equal); methodology (equal); project administration (equal); resources (equal); visualization (supporting); writing – original draft (supporting); writing – review and editing (equal). **David Rodriguez:** Funding acquisition (equal); project administration (supporting); resources (equal); supervision (equal); writing – review and editing (equal). **Shawn F. McCracken:** Conceptualization (equal); formal analysis (equal); funding acquisition (lead); investigation (equal); methodology (equal); project administration (lead); resources (equal); supervision (lead); validation (supporting); visualization (equal); writing – original draft (equal); writing – review and editing (lead).

## CONFLICT OF INTEREST

The authors declare no competing interests.

## Data Availability

The data and code from this manuscript are available on FigShare at https://doi.org/10.6084/m9.figshare.21382062.v1.
